# Использование гиперспектральной камеры Specim IQ для анализа растений

**DOI:** 10.18699/VJ19.587

**Published:** 2020-05

**Authors:** В.В. Альт, Т.А. Гурова, О.В. Елкин, Д.Н. Клименко, Л.В. Максимов, И.А. Пестунов, О.А. Дубровская, М.А. Генаев, Т.В. Эрст, К.А. Генаев, Е.Г. Комышев, В.К. Хлесткин, Д.А. Афонников

**Affiliations:** Сибирский федеральный научный центр агробиотехнологий Российской академии наук, р.п. Краснообск, Новосибирская область, Россия; Сибирский федеральный научный центр агробиотехнологий Российской академии наук, р.п. Краснообск, Новосибирская область, Россия; Сибирский федеральный научный центр агробиотехнологий Российской академии наук, р.п. Краснообск, Новосибирская область, Россия; Сибирский федеральный научный центр агробиотехнологий Российской академии наук, р.п. Краснообск, Новосибирская область, Россия; Институт автоматики и электрометрии Сибирского отделения Российской академии наук, Новосибирск, Россия; Институт вычислительных технологий Сибирского отделения Российской академии наук, Новосибирск, Россия; Институт вычислительных технологий Сибирского отделения Российской академии наук, Новосибирск, Россия; Федеральный исследовательский центр Институт цитологии и генетики Сибирского отделения Российской академии наук, Новосибирск, Россия Новосибирский национальный исследовательский государственный университет, Новосибирск, Россия; Федеральный исследовательский центр Институт цитологии и генетики Сибирского отделения Российской академии наук, Новосибирск, Россия; Федеральный исследовательский центр Институт цитологии и генетики Сибирского отделения Российской академии наук, Новосибирск, Россия; Федеральный исследовательский центр Институт цитологии и генетики Сибирского отделения Российской академии наук, Новосибирск, Россия; Федеральный исследовательский центр Институт цитологии и генетики Сибирского отделения Российской академии наук, Новосибирск, Россия; Федеральный исследовательский центр Институт цитологии и генетики Сибирского отделения Российской академии наук, Новосибирск, Россия Новосибирский национальный исследовательский государственный университет, Новосибирск, Россия

**Keywords:** hyperspectral data, spectral characteristics of plants, wheat diseases, root rot, potato pulp, chlorophyll, vegetation indices, гиперспектральные данные, спектральные характеристики растений, заболевания пшеницы, корневая гниль, мякоть картофеля, хлорофилл, вегетационные индексы

## Abstract

Важной технологией для неразрушающего мониторинга пигментного состава растений, который тесно связан с их физиологическим состоянием или заражением патогенами, является дистанционное зондирование при помощи гиперспектральных камер. В работе представлен опыт применения мобильной гиперспектральной камеры Specim IQ для исследований заболевания проростков четырех сортов пшеницы обыкновенной корневой гнилью (возбудитель – гриб Bipolaris sorokiniana Shoem.), а также для анализа мякоти клубней картофеля 82 линий и сортов. Для проростков были получены спектральные характеристики и по данным определены наиболее информативные спектральные признаки (индексы) для обнаружения корневой гнили. У проростков контрольных вариантов в видимой части спектра наблюдается возрастание отражательной способности с небольшим пиком в зеленой области (около 550 нм), затем идет понижение из-за поглощения света пигментами растений с экстремумом при длине волны около 680 нм. Анализ гистограмм значений вегетационных индексов показал, что индексы TVI и MCARI наиболее информативны для обнаружения патогена на проростках пшеницы по данным гиперспектральной съемки. Для образцов картофеля были выявлены участки спектра, соответствующие локальным максимумам и минимумам отражения. Показано, что спектры сортов картофеля имеют наибольшие различия в области длин волн 900–1000, 400–450 нм, что в первом случае может быть связано с уровнем содержания воды, а во втором – с формированием в клубнях меланина. По характеристикам спектра исследованные образцы разделились на три группы, каждая из которых содержит повышенные либо пониженные уровни интенсивности для указанных участков спектра. Кроме того, для ряда сортов были установлены минимумы в спектрах отражения, соответствующих хлорофиллу a. Результаты демонстрируют возможности камеры Specim IQ для проведения исследований гиперспектрального анализа растительных объектов.

## Abstract (en)

Remote sensing using hyperspectral cameras is an important technology for non-destructive monitoring of plant pigment composition, which is closely related to their physiological state or infection with pathogens. The paper presents the experience of using Specim IQ, a mobile hyperspectral camera, to study common root rot (the pathogen is the fungus Bipolaris sorokiniana Shoem.) affecting the seedlings of four wheat varieties and to analyze the pulp of potato tubers of 82 lines and varieties. Spectral characteristics were obtained for seedlings and the most informative spectral features (indices) for root rot detection were determined based on the data obtained. Seedlings of control variants in the visible part of the spectrum show an increase in reflectance with a small peak in the green area (about 550 nm), then a decrease due to light absorption by plant pigments with an extremum at a wavelength of about 680 nm. Analysis of histograms of vegetation index values demonstrated that the TVI and MCARI indices are the most informative for detecting the pathogen on wheat seedlings according to hyperspectral survey data. For potato samples, regions of the spectrum were found that correspond to local maxima and minima of reflection. It was shown that the spectra of potato varieties have the greatest differences within wavelength ranges of 900–1000 nm and 400–450 nm, which in the former case may be associated with the level of water content, and in the latter, with the formation of melanin in the tubers. It was shown that according to the characteristics of the spectrum, the samples studied are divided into three groups, each characterized by increased or reduced intensity levels for the specified parts of the spectrum. In addition, minima in the reflection spectra corresponding to chlorophyll a were found for a number of varieties. The results demonstrate the capabilities of the Specim IQ camera for conducting hyperspectral analyses of plant objects.

## Введение

Пигменты играют важную роль в жизни растений. Наиболее важные из них – хлорофиллы a и b, обеспечивающие процесс фотосинтеза, каротиноиды, ответственные за эффективность использования солнечной энергии при фотосинтезе, антоцианы, выполняющие защитные функции. Концентрации пигментов в растительных тканях могут быстро меняться в ответ на изменение физиологического состояния растений, и, таким образом, служить его маркерами. Состав и концентрацию пигментов можно исследовать химическими методами, однако более удобным способом, который активно развивается в последнее время, является метод дистанционной оценки на основе анализа спектров отраженного излучения (Мерзляк и др., 1997; Blackburn, 2007). Они основаны на том, что различные пигменты поглощают излучение разных длин волн неодинаково. В соответствии с этим у каждого пигмента есть свой характерный спектр отраженного излучения, который можно идентифицировать с использованием спектрометров высокого разрешения и интервала длин волн.

Развитие дистанционного мониторинга растений на основе мульти- и гиперспектральных сенсоров стало важным методом для оценки их физиологического состояния (Мерзляк и др., 2003), поражения заболеваниями (Mahlein et al., 2013), состояния плодов (Lorente et al., 2012) и корнеплодов (Rady et al., 2015; Pan et al., 2016). Такие сенсоры можно применять для оценки состояния растений как в лабораторных, так и в полевых условиях, а также устанавливать на беспилотные летательные аппараты (БПЛА) для широкого охвата посевов (Adão et al., 2017).

В настоящее время для получения мульти- и гиперспектральных изображений используют различные технические решения, которые отличаются технологией реализации сенсора, шириной спектрального интервала, спектральным и пространственным разрешением (Sellar, Boreman, 2005). Некоторые из них разработаны для установки на БПЛА, другие могут быть применены в лабораторных условиях. Однако все устройства требуют специальной технической настройки, установки и дополнительного оборудования для того, чтобы результаты можно было визуализировать. Гиперспектральная камера Specim IQ, применяемая в наших исследованиях, является компактным мобильным сенсором для наземной и лабораторной съемки (Bohnenkamp et al., 2018).

В нашей работе приведены результаты использования камеры Specim IQ для решения двух задач. Во-первых, проведено исследование проростков пшеницы четырех сортов в условиях заражения корневой гнилью и без заражения. Корневые гнили – наиболее вредоносные заболевания на пшенице в Западной Сибири. Из них существенное значение имеет гельминтоспориоз (возбудитель – гриб Bipolaris sorokiniana Shoem. = Drechslera sorokiniana Subram. et Jain, Helminthosporium sativum Pam.), поражающий практически все органы растения (пер- вичные, вторичные корни, колеоптиле, стебель, листья, зерно). Болезнь приводит к гибели всходов, отставанию в росте, отмиранию продуктивных стеблей, пустоколосице, щуплости зерна. Потери урожая в среднем составляют 15 % в результате снижения продуктивной кустистости, озерненности колоса и массы зерна (Долженко и др., 2014). Мы провели оценку информативности оптических параметров различных сортов пшеницы при действии возбудителя обыкновенной корневой гнили злаков B. sorokiniana Shoem.

Второй задачей было исследование спектральных характеристик мякоти клубней картофеля (Solanum tuberosum L.), представленных в коллекции Института цитологии и генетики СО РАН (ИЦиГ) «ГенАгро» (Новосибирск).

## Материалы и методы

**Образцы пшеницы для исследования поражения корневой гнилью.** Исследования проводили в лабораторных условиях (вегетационный опыт – водные культуры) на проростках районированных сортов мягкой яровой пшеницы селекции Сибирского научно-исследовательского института растениеводства – филиала ИЦиГ СО РАН: Новосибирская 18, Новосибирская 44, Сибирская 21 и Омского АНЦ – Омская 18.

Схема опыта включала варианты: контроль – семена без искусственной инфекции; опыт – семена искусственно инфицировали возбудителем обыкновенной гнили Bipolaris sorokiniana Shoem. (B. sorokiniana). Инфицирова- ние проросших семян осуществляли конидиальной суспензией смеси среднепатогенных изолятов B. sorokiniana, приготовленной на 0.1 % водном агаре (5000 конидий на 1 зерно). Для каждого сорта в вариантах закладывали по 100 зерен с учетом их всхожести. Инфекционную на- грузку наносили в капле суспензии возбудителя после подсчета конидий в камере Тома–Горяева. Отобранное, без внешних признаков поражения и повреждения зерно предварительно стерилизовали 90 % этиловым спиртом в течение 2 мин с последующим троекратным промыванием дистиллированной водой

Проростки выращивали в рулонной культуре на водопроводной воде в камере искусственного микроклимата «Биотрон-7» (разработка Сибирского физико-технического института (Сибирский федеральный научный центр агробиотехнологий Российской академии наук)) до фазы 1–2 листа при 16-часовом фотопериоде с освещенностью 20 000 лк (день), температура днем 22 °С, ночью – 18 °С, влажность – 60 % (Гурова и др., 2017). Для проведения съемки с помощью гиперспектральной камеры использовали побеги проростков пшеницы с признаками поражения корневой гнилью (штрихи и полосы темно-бурого цвета).

Образцы клубней картофеля. В исследовании использованы 82 образца картофеля (S. tuberosum L.) из коллек- ции ИЦиГ «ГенАгро» (Новосибирск). Все сорта и гибри- ды, анализируемые в ходе эксперимента, были выращены в полевых условиях (в пределах одной локации) с июня по начало сентября 2018 г. Все образцы были посажены одновременно или с разницей в один день и выкопаны таким же образом. Клубни всех образцов были посажены в два ряда с расстоянием между рядами в 0.75 м и между растениями – в 0.3 м. Длина каждого ряда составляла 3 м, выращивалось по 10 растений. После извлечения клубней из почвы их направляли на хранение в течение 3 нед при температуре 4 °С. По прошествии указанного времени отбирали только визуально здоровые, типичные по форме и размеру клубни для каждой линии. Клубни (два на образец) были вымыты водопроводной водой и оставлены на ночь для испарения водопроводной воды с поверхности клубней в комнатных условиях.

Для проведения съемки с помощью гиперспектральной камеры использовали срезы клубней. Выполняли поперечные разрезы клубней по центру с помощью ножа на две приблизительно равные доли, от одной из них проводился срез, максимально однородный по толщине в различных частях среза. Все срезы имели толщину в пределах 2–3 cм. В случаях невозможности выполнения поперечного среза по центру клубня (выявляемые дефекты только при разрезании клубня, например, такие, как результат поражения различными инфекциями) делали продольный срез по центру доли клубня, полученной припоперечном разрезе. Срезы в пределах изучаемого сорта или гибрида выполняли непосредственно перед съемкой.

Получение гиперспектральных изображений. Спектральные характеристики проростков пшеницы и клубней картофеля для анализа были собраны с помощью гипер- спектральной камеры Specim IQ (Spectral Imaging Ltd., https://www.specim.fi/iq/). Эта камера позволяет оценивать спектры отражения в интервале 400–1000 нм. Спектральное разрешение камеры составляет 7 нм и включает 204 полосы, пространственное разрешение сенсора – 512 × 512 пикселей. Технические характеристики камеры более подробно приведены в работе (Bohnenkamp et al., 2018). Камера была любезна предоставлена ООО «Компания «Азимут Фотоникс» (Москва). Камера была установлена на штативе над столом, на расстоянии 15–20 см от образца, расположенного на белом листе бумаги. Образцы освещались тремя галогенными лампами: две мощностью 500 Вт, одна – 700 Вт, как это рекомендовано в инструкции к камере. Перед съемкой проводилась калибровка камеры с помощью калибровочной панели, после этого при получении серий снимков панель удаляли из кадра.

Обработка спектральных изображений. Предварительный визуальный контроль качества гиперспектральных изображений осуществлялся с помощью программы Specim IQ Studio software. Для массовой обработки изображений использовали библиотеки языка Python. Извлечение данных по интенсивности спектральных линий из выходных файлов Specim IQ в формате envi проводили с помощью пакета spectral (http://www.spectralpython.net/). Сглаживание спектров было выполнено с использованием фильтра Савицкого–Голая (Savitzky, Golay, 1964), программа savgol_filter – из пакета scipy (https://www.scipy.org/).

Выделение областей проростков и срезов клубней осуществляли на основе анализа интенсивности сигнала по различным линиям спектра (белый фон имел практически одинаковую интенсивность отражения по всем линиям спектра).

Для исследования были взяты от трех до пяти гиперспектральных изображений здоровых и зараженных проростков для каждого сорта пшеницы. При получении спектральных кривых проводили сегментацию изображений и использовали средние значения спектральных яркостей выделенных сегментов по нескольким изображениям. При анализе проростков рассчитывали серию вегетационных индексов, как это было описано ранее в работе (Дубровская и др., 2018).

При анализе клубней для каждого изображения мы выбирали случайным образом 6000 пикселей (выборка с возвращением), принадлежащих области мякоти клубня, и по ним усредняли значение интенсивностей для каждой линии спектра. Полученные таким образом средние значения характеризовали спектры каждого из 82 образцов мякоти картофеля.

Для предсказания содержания крахмала и мезги в клубнях картофеля по гиперспекральным данным применен метод частных наименьших квадратов (PLS), в качестве зависимых переменных рассматривали значения содержания крахмала или мезги, независимыми переменными были значения первой производной интенсивностей спектра (дифференциальные кривые).

## Результаты

Анализ данных по корневой гнили пшеницы. Анализ спектральных кривых, полученных для проростков сортов Новосибирская 18, Омская 18, Новосибирская 44 и Сибирская 21, показал, что отражательные характеристики здоровых проростков пшеницы и инфицированных возбу- дителем обыкновенной корневой гнили злаков отличаются в исследуемых частях спектра – видимой (400–700 нм) и ближней инфракрасной области (700–900 нм) (рис. 1). У проростков контрольных вариантов в видимой части спектра наблюдается возрастание отражательной способности с небольшим пиком в зеленой области (около 550 нм), затем идет понижение из-за поглощения света пигментами растений с экстремумом при длине волны около 680 нм. В ближней инфракрасной области отражательная способность проростков контрольных и опытных вариантов повышается, что связано с внутренним рассеянием света мезофиллом (Behmann et al., 2014).

**Fig. 1.**
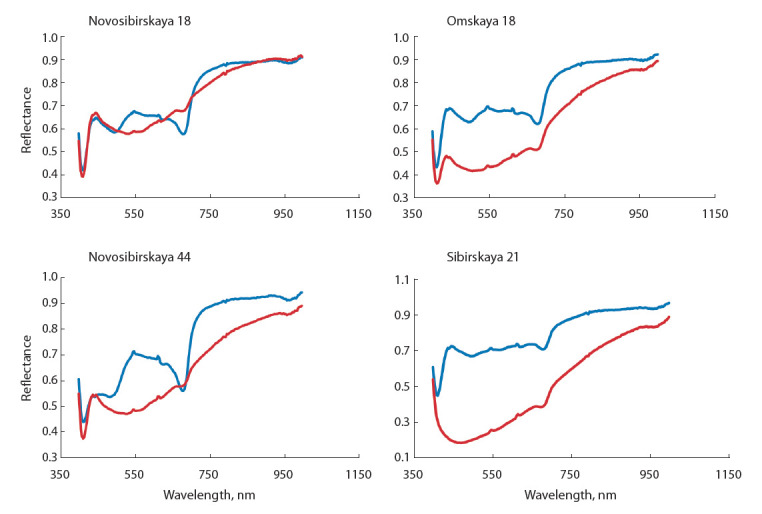
Spectra of wheat varieties: blue curve, healthy seedlings; red curve, seedlings damaged by wheat scab (B. sorokiniana).

Различия отражательных характеристик в определен- ных зонах спектра послужили основой для применения вегетационных индексов для обнаружения и диагностики корневой гнили на посевах и распознавания особенностей здоровых и пораженных заболеванием всходов пшеницы. В результате анализа различных вегетационных индексов, ранее используемых при диагностике и мониторинге развития других заболеваний пшеницы (Дубровская и др., 2018), а также на основе анализа спектральных характеристик, полученных при лабораторном эксперименте, было выбрано 13 вегетационных индексов для идентификации корневой гнили (В. sorokiniana) (табл. 1). Детальное описание индексов приведено в работе (Дубровская и др., 2018).

**Table 1.**
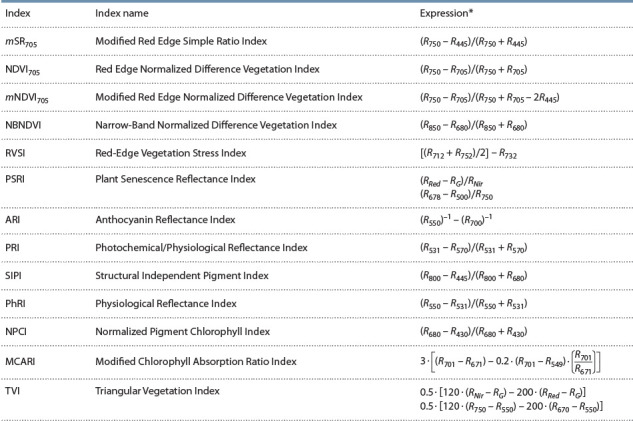
Vegetation indices used for wheat scab identification on wheat seedlings RX – is reflectance at the corresponding wavelength or in the corresponding spectral range: G (green – 520–600 nm), Red (630–690 nm), Nir (760–900 nm).

Анализ гистограмм значений вегетационных индексов показал (рис. 2), что индексы TVI и MCARI наиболее информативны для обнаружения патогена на проростках пшеницы, по данным гиперспектральной съемки.

**Fig. 2.**
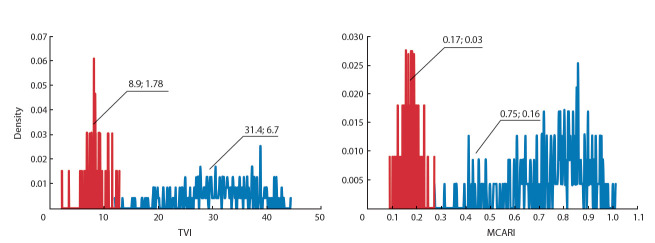
Imputed distribution densities of the indices TVI (left) and MCARI (right) for classes of seedlings (cv. Novosibirskaya 18): blue bars, healthy plants; red bars, plants damaged by wheat scab. Callouts indicate sample means and standard deviations of indices.

Анализ мякоти клубней картофеля. Спектральные кривые мякоти клубней 82 образцов картофеля пред- ставлены на рис. 3, а.

**Fig. 3.**
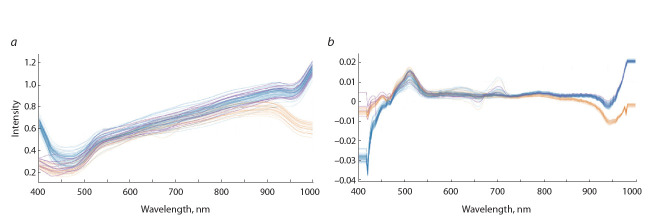
Spectral features of potato tuber flesh in 82 potato genotypes: a – mean reflection intensities for 204 spectral lines within 400–1000 nm; b – differential curves. Colors of the curves correspond to three types of spectral lines (see text).

Из диаграмм видны две характерные спектральные области, в которых наблюдаются существенные различия в интенсивности спектров отражения различных сортов: 420–470 и 860–980 нм. Для области 420–470 нм наблюдаются два характерных типа значений интенсивности: больше 0.4 (53 образца, 64 %) и меньше этого значения (см. рис. 3, a; 30 образцов, 36 %). Для области 860–980 нм также наблюдаются два типа значений: больше 0.8 (см. рис. 3, а; 63 образца, 75 %) и меньше этого значения (20 образцов, 25 %). Более детальный анализ показал, что все образцы в выборке можно разделить на три группы по значению величин интенсивности спектра в этих двух областях. К первой группе (А) относятся образцы, у которых значение интенсивности в интервале 420–470 нм больше 0.4, а в интервале 860–980 нм – больше 0.8. Таких образцов оказалось 53 (на рис. 3 они показаны синим цветом). Списки названий сортов картофеля, которые относятся к этой и двум другим группам, приведены в Приложении 1)^1^ поглощения которых в интервале 420–470 нм меньше 0.4, а в интервале 860–980 нм – меньше 0.8. Таких образцов оказалось 20 (на рис. 3 они выделены оранжевым цветом). К группе С относятся образцы, у которых интенсивность поглощения в области 420–470 нм меньше 0.4, а в области 860–980 нм – больше 0.8. Таких образцов было 9 (на рис. 3 они показаны сиреневым цветом). Эти три кластера оказались хорошо различимы на графике рассеяния для двух главных компонент, полученных при анализе производных от спектров (рис. 4). Образцов, у которых в области спектра 420–470 нм интенсивность больше 0.4, а в области 860–980 нм – меньше 0.8, не обнаружено.

Приложения 1 и 2 см. по адресу: http://www.bionet.nsc.ru/vogis/download/pict-2020-24/appx2.pdf



**Fig. 4.**
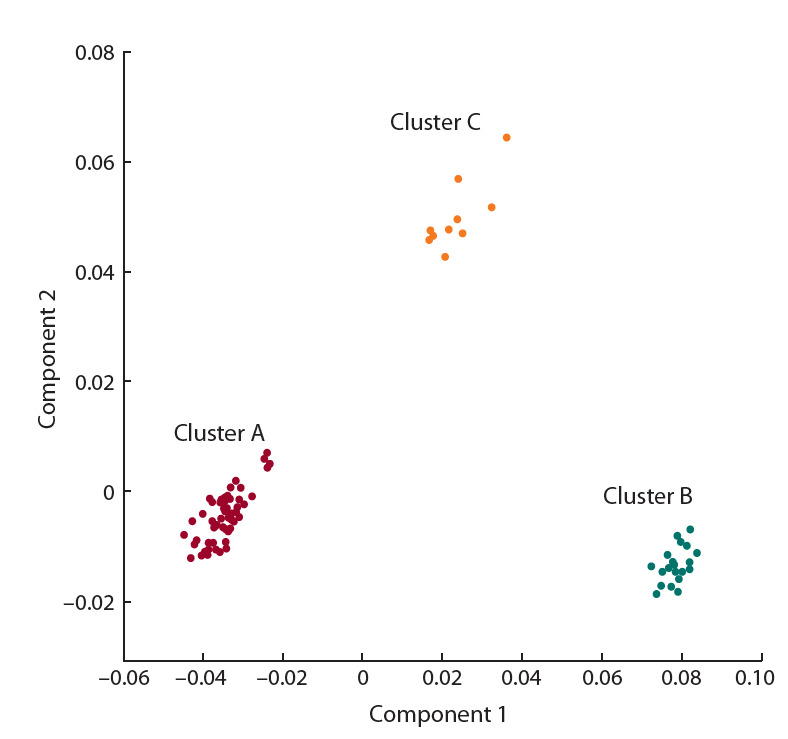
Scattering of two principal components in 82 specimens obtained by principal component analysis of differential curves. X-axis: component 1 (79.9 % of variation); Y-axis: component 2 (12.9 % of variation).

Необходимо отметить, что эти кластеры также можно идентифицировать на основе анализа диаграммы рассеяния в пространстве двух главных компонент для интенсивностей спектральных линий пикселей, соответ- ствующих областям мякоти клубней (Приложение 2). На этой диаграмме также видно, что пиксели клубней одного сорта формируют компактные облака, которые хорошо отделяются от областей, соответствующих другим генотипам картофеля.

В спектрах некоторых образцов наблюдаются менее выраженные различия в области 640–730 нм. Этот интервал длин волн соответствует области поглощения света хлорофиллом (Guidi et al., 2017).

## Обсуждение

Проведенные нами исследования показали возможность применения камеры Specim IQ для анализа спектров отражения растительных образцов (проростков пшеницы и срезов клубней картофеля). В первом случае удалось выявить значимые различия в спектрах проростков здоровых и пораженных корневой гнилью. У здоровых проростков минимумы отражения наблюдаются в полосах сильного поглощения хлорофиллов в красной области спектра (680 нм) и в полосе совместного поглощения хлорофиллов и каротиноидов в синей области (~500 нм).

У пораженных проростков трех сортов пшеницы (кроме сорта Новосибирская 18) коэффициенты отражения практически во всем диапазоне значительно ниже, чем у здоровых. Снижение отражательной способности у пораженных проростков, наиболее выраженное у сорта Си- бирская 21, возможно, обусловлено особенностями протекания инфекционного процесса и формирования защитно-приспособительных реакций при патогенезе.

Резкое изменение спектральных характеристик растений на границе видимой красной и ближней инфракрас- ной частей спектра в диапазоне 690…740 нм (положение «красных краев») имеет большое значение для диагностики стрессовых воздействий. Подобную особенность отмечали A. Lowe с коллегами (2017). Она может быть объяснена тем, что хлорофилл сильно поглощает длины волн вплоть до 700 нм, и, следовательно, растительный материал в этом диапазоне имеет низкую отражательную способность, которая резко возрастает в ближней инфракрасной области спектра (~720 нм). Обнаружено, что различия в спектрах можно описать за счет изменения ряда известных индексов, в частности TVI и MCARI, связанных с содержанием хлорофилла.

При анализе срезов клубней картофеля мы выделили две области спектра (400–450 и 900–1000 нм), в которых наблюдаются наибольшие различия в нашей выборке об разцов. Их можно связать с областями поглощения ряда растительных пигментов, а также молекул воды и групп OH (табл. 2).

**Table 2.**
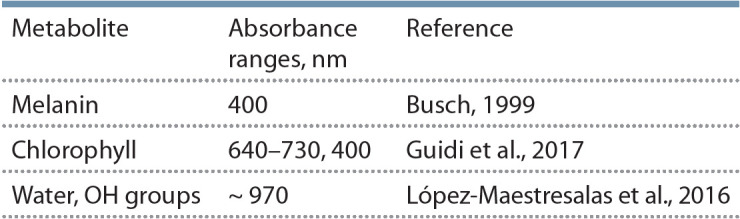
Absorbance ranges of some metabolites

Обнаруженные полосы поглощения позволяют предположить наличие или образование в ходе эксперимента (регистрации спектра) поглощающих в видимой или ближней инфракрасной области спектра метаболитов. Так, известно, что в овощах при взаимодействии производных тирозина с кислородом воздуха в присутствии оксидаз может происходить образование меланина – олигомерного окрашенного соединения (Busch, 1999), имеющего в спектре полосу поглощения при 400 нм. Возможно, в процессе экспонирования некоторые сорта картофеля более легко подвергались каталитическому окислению с образованием коричневой окраски.

Поглощение в области 640–730 нм может соответствовать наличию зеленого пигмента – хлорофилла – в краевых областях срезов в некоторых рассмотренных образцах картофеля (Tanios et al., 2018). Обе распространенные в растениях разновидности хлорофилла – a и b – поглощают свет 640–730 нм. Кроме того, хлорофилл a имеет полосу поглощения при 400 нм (Guidi et al., 2017). Возможно, именно сорта с хлорофиллом в краевых участках срезов клубней относятся к группе С по РСА (см. рис. 4

Поглощение при 860–980 нм относится к водородным связям воды (López-Maestresalas et al., 2016) и, возможно, связанных с ней полярных полимеров (крахмала, пектинов, целлюлозы), присутствующих в мякоти картофеля. Различием в связывании воды и групп ОН можно объяснить наличие двух групп сортов картофеля.

Оценка скорости потемнения среза клубня (образования мелатонина) имеет практическое значение при производстве чипсов и полупродуктов для жареного картофеля. Анализ наличия хлорофилла в картофеле позволяет судить о том, экспонировался ли картофель на свету и, соответственно, есть ли риск присутствия в нем ядовитого алкалоида соланина. Таким образом, использование гиперспектральной камеры имеет значительный потенциал для оценки наличия метаболитов в картофеле по его срезу и автоматизации процессов переработки картофеля в продукты с высокой добавленной стоимостью.

## Заключение

С помощью гиперспектральной камеры Specim IQ проведен анализ двух типов биологических образцов: проростков пшеницы четырех сортов с поражением корневой гнилью (возбудитель – B. sorokiniana Shoem.) и здоровых. Выявлено наличие различий в спектрах здоровых и пораженных растений, определены индексы TVI и MCARI, которые в наибольшей степени различаются для здоровых и больных проростков. Анализ мякоти клубней картофеля показал выраженные группы образцов, которые различаются по интенсивности спектров отражения в областях 400–450 и 900–1000 нм, что может быть связано с областями поглощения меланина в первом случае (почернение мякоти) и молекул воды и групп OH – во втором (содержание влаги).

## Conflict of interest

The authors declare no conflict of interest.

## References

Гурова Т.А., Денисюк С.Г., Луговская О.С., Свежинцева Е.А., Ми-
неев В.В. Методические положения ранней диагностики устой-
чивости сортов яровой пшеницы и ячменя к совокупному дей-
ствию стрессоров. Новосибирск: СФНЦА РАН, 2017.
[Gurova T.A., Denisyuk S.G., Lugovskaya O.S., Svezhintseva E.A.,
Mineev V.V. Methodological Provisions for Early Diagnostics of
Spring Wheat and Barley Varieties Resistance to the Combined Action
of Stressors. Novosibirsk, 2017. (in Russian)]

Долженко В.И., Власенко Н.Г., Власенко А.Н., Коротких Н.А., Теплякова
О.И., Кулагин О.В., Слободчиков А.А., Кудашкин П.И.,
Любимец Ю.В., Гаркуша А.А., Стецов Г.Я., Садовников Г.Г., Са-
довникова Н.Н., Бочарова Л.С., Доронин В.Г., Тимофеев В.Н.,
Гарбар Л.И. Зональные системы защиты яровой пшеницы от
сорняков, болезней и вредителей в Западной Сибири. Новоси-
бирск: ГНУ СибНИИЗиХ, 2014.
[Dolzhenko V.I., Vlasenko N.G., Vlasenko A.N., Korotkikh N.A.,
Teplyakova O.I., Kulagin O.V., Slobodchikov A.A., Kudashkin P.I.,
Lyubimets Y.V., Garkusha A.A., Stetsov G.Ya., Sadovnikov G.G.,
Sadovnikova N.N., Bocharova L.S., Doronin V.G., Timofeev V.N.,
Garbar L.I. Zonal Systems of Spring Wheat Protection from Weeds,
Diseases, and Pests in Western Siberia. Novosibirsk, 2014. (in Russian)]

Дубровская О.А., Гурова Т.А., Пестунов И.А., Котов К.Ю. Методы
обнаружения болезней на посевах пшеницы по данным дистан-
ционного зондирования (обзор). Сиб. вестн. с.-х. науки. 2018;
48(6):76-89. DOI 10.26898/0370-8799-2018-6-11.
[Dubrovskaya O.A., Gurova T.A., Pestunov I.A., Kotov K.Yu.
Methods of detection of diseases on wheat crops according to remote
sensing (overview). Sibirskiy Vestnik Selskokhozyaystvennoy
Nauki = Siberian Herald of Agricultural Sciences. 2018;48(6):76-
89. DOI 10.26898/0370-8799-2018-6-11. (in Russian)]

Мерзляк М.Н., Гительсон А.А., Чивкунова О.Б., Соловченко А.Е.,
Погосян С.И. Использование спектроскопии отражения в ана-
лизе пигментов высших растений. Физиол. растений. 2003;
50(5):785-792.
[Merzlyak M.N., Gitelson A.A., Chivkunova O.B., Solovchenko
A.E., Pogosyan S.I. Application of reflectance spectroscopy for
analysis of higher plant pigments. Russ. J. Plant Physiol. 2003;50:
704-710. DOI 10.1023/A:1025608728405.]

Мерзляк М.Н., Чивкунова О.Б., Гительзон А.А., Погосян С.И., Ле-
химена Л., Гарсон М., Бузулукова Н.П., Шевырева В.В., Румян-
цева В.Б. Спектры отражения листьев и плодов при их разви-
тии, старении и стрессе. Физиол. растений. 1997;44(5):707-716.
[Merzlyak M.N., Chivkunova O.B., Gitelzon A.A., Pogosyan S.I.,
Lejimena L., Garson M., Buzulyukova N.P., Shevyreva V.V., Rumyantseva
V.B. Reflection spectra of leaves and fruit during their
development, aging, and stress. Fiziologiya Rasteniy = Plant Physiology.
1997;44(5):707-716. (in Russian)]

Adão T., Hruška J., Pádua L., Bessa J., Peres E., Morais R., Sousa J.
Hyperspectral imaging: A review on UAV-based sensors, data processing
and applications for agriculture and forestry. Remote Sens.
2017;9(11):1110.

Behmann J., Steinrücken J., Plümer L. Detection of early plant stress
responses in hyperspectral images. ISPRS J. Photogramm. Remote
Sens. 2014;93:98-111. DOI 10.1016/j.isprsjprs.2014.03.016.

Blackburn G.A. Hyperspectral remote sensing of plant pigments.
J. Exp. Bot. 2007;58(4):855-867.

Bohnenkamp D., Kuska M.T., Jussila J., Salo H., Mahlein A.-K.,
Rasche
U. Specim IQ: evaluation of a new, miniaturized handheld hyperspectral
camera and its application for plant phenotyping and
disease detection. Sensors. 2018;18(2):441.

Busch J.M. Enzymic browning in potatoes: a simple assay for a polyphenol
oxidase catalysed reaction. Biochem. Educ. 1999;27(3):
171-173.

Guidi L., Tattini M., Landi M. How does chloroplast protect chlorophyll
against excessive light? In: Jacob-Lopes E., Queiroz Zepka L.,
Queiroz M.I. (Eds.). Chlorophyll. 2017;21. DOI 10.5772/67887.

López-Maestresalas A., Keresztes J.C., Goodarzi M., Arazuri S.,
Jarén C., Saeys W. Non-destructive detection of blackspot in potatoes
by Vis-NIR and SWIR hyperspectral imaging. Food Control.
2016;70:229-241.

Lorente D., Aleixos N., Gómez-Sanchis J.U.A.N., Cubero S., García-
Navarrete O.L., Blasco J. Recent advances and applications of hyperspectral
imaging for fruit and vegetable quality assessment. Food
Bioproc. Technol. 2012;5(4):1121-1142.

Lowe A., Harrison N., French A.P. Hyperspectral image analysis techniques
for the detection and classification of the early onset of plant
disease and stress. Plant Methods. 2017;13:2-12.

Mahlein A.K., Rumpf T., Welke P., Dehne H.W., Plümer L., Steiner U.,
Oerke E.C. Development of spectral indices for detecting and identifying
plant diseases. Remote Sens. Environ. 2013;128;21-30.

Pan L., Lu R., Zhu Q., Tu K., Cen H. Predict compositions and mechanical
properties of sugar beet using hyperspectral scattering.
Food Bioprocess Technol. 2016;9(7):1177-1186.

Rady A., Guyer D., Lu R. Evaluation of sugar content of potatoes using
hyperspectral imaging. Food Bioprocess Technol. 2015;8(5):995-
1010.

Savitzky A., Golay M.J. Smoothing and differentiation of data by
simplified least squares procedures. Anal. Сhem. 1964;36(8):1627-
1639.

Sellar R.G., Boreman G.D. Classification of imaging spectrometers for
remote sensing applications. Opt. Eng. 2005;44(1):013602.
Tanios S., Eyles A., Tegg R., Wilson C. Potato tuber greening: a review
of predisposing factors, management and future challenges. Am. J.
Potato Res. 2018;95(3):248-257.

